# *Lacticaseibacillus rhamnosus* D1 Fermented Milk Confers Protection Against Typhoid Fever Through Immunomodulation and Gut Microbiota Regulation in Mice

**DOI:** 10.3390/microorganisms13102348

**Published:** 2025-10-14

**Authors:** Leonardo Acurcio, Sávio Sandes, Diego Rios, Felipe Sant’Anna, Silvia Pedroso, Rafael Bastos, Marcelo Souza, Jacques Nicoli

**Affiliations:** 1 Departamento de Microbiologia, Instituto de Ciências Biológicas, Universidade Federal de Minas Gerais (UFMG), Avenida Antônio Carlos, n° 6627, Bairro Pampulha, Belo Horizonte 31270-901, MG, Brazil; leoacurcio@gmail.com (L.A.); shspietra@gmail.com (S.P.); rafawbastos@gmail.com (R.B.); jnicoli@icb.ufmg.br (J.N.); 2 Centro Universitário de Formiga (UNIFOR-MG), Rua Dr. Arnaldo Sena, n° 328, Bairro Água Vermelha, Formiga 35570-000, MG, Brazil; 3 Departamento de Genética, Ecologia e Evolução, Instituto de Ciências Biológicas, Universidade Federal de Minas Gerais (UFMG), Avenida Antônio Carlos, n° 6627, Bairro Pampulha, Belo Horizonte 31270-901, MG, Brazil; diego.rios@ifam.edu.br; 4 Departamento de Ciências de Alimentos e Nutrição, Faculdade de Engenharia de Alimentos, Universidade Estadual de Campinas (UNICAMP), Rua Monteiro Lobato, n° 80, Bairro Cidade Universitária, Campinas 13083-862, SP, Brazil; 5 Campus São Gabriel da Cachoeira, Instituto Federal do Amazonas (IFAM), BR 307, Km 03, s/n°, Bairro Cachoeirinha, São Gabriel da Cachoeira 69750-000, AM, Brazil; 6 Departamento de Tecnologia e Inspeção de Produtos de Origem Animal, Escola de Veterinária, Universidade Federal de Minas Gerais (UFMG), Avenida Antônio Carlos, n° 6627, Bairro Pampulha, Belo Horizonte 31270-901, MG, Brazil; vetfelipem@gmail.com (F.S.); marceloresende51@gmail.com (M.S.); 7 College of Agriculture and Life Sciences, University of Vermont (UVM), 570, Main Street, Burlington, VT 05405, USA; 8 Departamento de Antibióticos, Centro de Biociências, Universidade Federal de Pernambuco (UFPE), Av. Prof. Moraes Rego, n° 1235, Bairro Cidade Universitária, Recife 50670-901, PE, Brazil; 9 Departamento de Microbiologia e Parasitologia, Centro de Biociências, Universidade Federal do Rio Grande do Norte (UFRN), Avenida Senador Salgado Filho, n° 3000, Bairro Lagoa Nova, Natal 59064-741, RN, Brazil

**Keywords:** fermented milk, *Lacticaseibacillus rhamnosus* D1, *Salmonella* Typhimurium, immunomodulation, antimicrobial peptides, gut microbiota

## Abstract

This study investigated the protective effect of fermented milk by *Lacticaseibacillus rhamnosus* D1 in a murine model of Typhoid fever, focusing on cytokines, antimicrobial peptides and microbiota modulation. BALB/c mice were pre-treated with milk fermented by *L. rhamnosus* D1 prior to *Salmonella* Typhimurium challenge. Outcomes assessed included survival, weight change, bacterial translocation, mRNA expression of cytokines and antimicrobial peptides, in addition to gut microbiota modulation. Mice receiving fermented milk exhibited higher survival rates, reduced bacterial translocation and attenuated weight loss compared to controls. mRNA expression analyses revealed that *L. rhamnosus* D1 pre-treatment suppressed the expression of pro-inflammatory cytokines (IFN-γ, IL-6 and IL-12) and upregulated anti-inflammatory cytokines (IL-5, IL-10 and TGF-β), as well as antimicrobial peptides (Reg3β, Reg3γ and Lcn2). Furthermore, we observed that the consumption of fermented milk changed the gut microbiota of infected mice, not only by modulating the existing taxa, but also by facilitating the emergence of unique, potentially beneficial microbial lineages, such as *Muribaculum*, *Roseburia*, *Intestinimonas*, *Bdellovibrio* and *Facklamia*. These findings indicate that *L. rhamnosus* D1 protected mice against *S.* Typhimurium infection through immunomodulatory and microbiota-mediated mechanisms, changing mucosal immunity and strengthening the intestinal barrier by modulating gut microbiota and immune responses, in addition to promoting host antimicrobial defenses.

## 1. Introduction

Typhoid fever is an infectious disease caused by *Salmonella enterica* serovar Typhi, a Gram-negative, facultative intracellular pathogenic bacteria [1,2]. Transmission occurs primarily through the ingestion of water or food contaminated with the feces or urine of infected individuals, characterizing it as a fecal–oral route disease [2]. Once ingested, *S.* Typhi is able to resist gastric acidity, reaching the small intestine, where it establishes itself and proliferates, causing inflammation and damage to intestinal mucosa (Local Phase). Additionally, it has the ability to disrupt the intestinal epithelial barrier, migrating to mesenteric lymph nodes and disseminating via the bloodstream to reticuloendothelial organs such as the liver, spleen and bone marrow (Systemic Phase, [3,4,5,6,7]). In the host, *S.* Typhi infection may manifest with nonspecific systemic symptoms, including a prolonged high-grade fever, malaise, abdominal pain and bacteremia [3,6,7]. In severe cases, it may lead to intestinal perforation, hemorrhage and septic shock, with a significant risk of mortality if left untreated [7]. From an epidemiological point of view, Typhoid fever remains a significant public health concern in low- and middle-income countries. Global estimates suggest that 9 to 12 million cases occur annually, resulting in approximately 110,000 to 160,000 deaths worldwide [3,8,9]. The disease is endemic in regions with an inadequate access to safe water, sanitation and hygiene infrastructure, particularly in South Asia, Southeast Asia and parts of Sub-Saharan Africa [4,7]. Antimicrobial therapy is the mainstay of treatment, with agents such as ciprofloxacin, azithromycin or ceftriaxone commonly prescribed in non-complicated cases [7,8,10]. However, the emergence and spread of multidrug-resistant (MDR) and extensively drug-resistant (XDR) strains of *S.* Typhi have posed a significant challenge to effective treatment [8,10].

An alternative strategy within this epidemiological context may involve the application of probiotics which, through their beneficial interactions with the host, could contribute to the reinforcement of the intestinal epithelial barrier, preventing the progression of the Systemic Phase of Typhoid fever and, consequently, severe clinical manifestations [2,11,12]. Through several mechanisms, these beneficial microorganisms can modulate the intestinal mucosa by influencing inflammatory signaling pathways, enhancing mucus secretion and antimicrobial peptide production, as well as competing with pathogenic microorganisms—including *S.* Typhi—for nutrients and adhesion sites [2,13,14]. Furthermore, probiotics are able to modulate the composition and functionality of the gut microbiota, counteracting dysbiosis and promoting the restoration of microbial homeostasis characterized by an increased species richness and diversity [15,16,17]. Among the diverse range of probiotic strains, *Lacticaseibacillus rhamnosus* GG stands out as one of the most extensively studied, with a wealth of evidence supporting its beneficial effects on various aspects of human health, including the prevention and treatment of gastrointestinal disorders, the modulation of immune responses and the alleviation of allergic symptoms [18]. The precise mechanisms by which *L. rhamnosus* GG exerts its protective effects are multifaceted and involve complex interactions with the host’s immune system, the gut microbiota and the intestinal epithelial cells. Notably, lactobacilli members have been proposed to develop symbiotic relationships that can promote cell proliferation and migration, facilitate healing and modify epithelial cytokine signaling [13,19]. Understanding these mechanisms is crucial for developing targeted probiotic interventions that can effectively promote health and prevent disease.

The efficacy of probiotics in improving intestinal health can vary based on factors such as dose, strain and individual characteristics, necessitating customized approaches for maximum benefits. Moreover, the capacity of probiotics to modulate cytokine production and defensin expression highlights their potential as therapeutic agents [11,12,20]. Cytokines play a pivotal role in orchestrating immune responses, with different cytokines promoting either pro-inflammatory or anti-inflammatory effects. Probiotics can influence the balance of cytokine production, promoting the secretion of anti-inflammatory cytokines, such as IL-10 and TGF-β, while suppressing the production of pro-inflammatory cytokines, such as TNF-α and IL-6 [14,21]. This modulation of cytokine production can help to dampen excessive inflammatory responses and restore immune homeostasis, and may play an important role in sepsis caused by *S.* Typhi [11,12,22]. Additionally, probiotics can stimulate the expression of antimicrobial peptides that are produced by epithelial cells and immune cells, providing an additional layer of defense against invading pathogens [23,24,25].

In light of these considerations, we evaluated the protective effect of milk fermented by *Lacticaseibacillus rhamnosus* D1 in a murine model of Typhoid fever, whose infection caused by *S.* Typhimurium resembles that generated by *S.* Typhi in humans [11,12]. *L. rhamnosus* D1, isolated from Minas Artisanal Cheese produced in the Serra da Canastra region (Brazil), has demonstrated promising functional and probiotic features in previous studies (unpublished data). These include the ability to survive to gastrointestinal transit and exert antagonistic activity against pathogenic bacteria such as *Escherichia coli*, *Salmonella enterica* and *Listeria monocytogenes*, among others. Therefore, we investigated whether the consumption of milk fermented by this strain confers protection to mice against infections caused by *S.* Typhimurium. For this purpose, we analyzed physiological, histopathological, immunological and microbiological parameters. The results of this study contributed to clarifying the potential mechanisms associated with the beneficial effects of beverage consumption and demonstrated its potential as a therapeutic intervention for Typhoid fever in humans.

## 2. Materials and Methods

### 2.1. Bacterial Strains, Culture Conditions and Fermented Milk

*Lacticaseibacillus rhamnosus* D1 was isolated from Minas Artisanal Cheese in a previous study [26]. The strain was maintained in De Man, Rogosa and Sharpe (MRS–Merck Millipore, Darmstadt, Germany) broth supplemented with 20% glycerol (*v*/*v*) at −80 °C. For administration to mice, fermented milk was prepared as follows: (1) frozen strain was inoculated in MRS broth (2% *v*/*v* inoculum) and cultivated at 37 °C for 24 h until stationary phase; (2) after this period, sterile milk solution was inoculated with activated *L. rhamnosus* D1 (2% *v*/*v* inoculum). This milk solution consisted of skim milk powder 10% (*w*/*v*, Molico^®^, Nestlé Brasil Ltda., São Paulo, SP, Brazil) and sucrose 10% (*w*/*v*) P.A.-A.C.S. (Synth, Diadema, SP, Brazil) in distilled water. Fermentation lasted 12 h at 37 °C, which was the time required to reach the minimum count of 10^8^ colony formation units per mL (CFU/mL). *L. rhamnosus* D1 counts throughout the experimental period were assessed every week by serial dilution and plating on MRS agar (Merck Millipore, Darmstadt, Germany) to ensure that a minimum count of 10^8^ CFU/mL was preserved throughout the experimental period.

*S.* Typhimurium was obtained through culture collection from the Laboratory of Ecology and Physiology of Microorganisms (LEFM/ICB/UFMG, Belo Horizonte, Brazil) and was cultivated in brain heart infusion broth (BHI, Neogen, Lansing, MI, USA) at 37 °C for 18 h under aerobic conditions. Our research group frequently uses this *Salmonella* strain in mice infections, which is known to be fatal to mice and causes an enteritis mimicking the disease caused by *S.* Typhi in humans [11,12].

### 2.2. Mice

Four-week-old male BALB/c mice were supplied by the Centre for Animal Care (CEBIO) of the Federal University of Minas Gerais (UFMG), Belo Horizonte, Brazil. Animals were maintained in a ventilated animal caging system (Alesco, Monte Mor, Brazil) with controlled lighting (12 h light–dark cycle), humidity (60–80%) and temperature (22 ± 1 °C).

Water and a commercial autoclavable diet (Nuvital, Nuvilab CR1, Curitiba, Brazil) were sterilized by steam and administered ad libitum. All experimental procedures were carried out according to the Brazilian National Council for the Control of Animal Experimentation (CONCEA), and are in accordance with the guidelines for the care and use of laboratory animals described by the US National Institutes of Health. We acknowledge our compliance with these guidelines. The Ethics Committee in Animal Experimentation (CEUA) from UFMG approved the study (Protocol numbers 380/2013 and 351/2016).

### 2.3. Treatment, Typhoid Fever Induction and Sample Collection

Treatment with milk fermented by *L. rhamnosus* D1 was conducted daily, for 7 or 15 days prior to *S.* Typhimurium infection (see below). Experimental mice received, by oral gavage, 0.1 mL of fermented milk containing 10^8^ CFU/mL of *L. rhamnosus* D1, being the minimum dose at which its beneficial effects could be observed. The treatment continued daily until the end of experiment. Mice were single-infected by oral gavage with 0.1 mL of *S.* Typhimurium suspension containing 10^6^ CFU/mL [11].

In the first set of experiments (Mortality Assay), we evaluated the protective effect of milk fermented by *L. rhamnosus* D1 on the mortality rate of mice infected with *S.* Typhimurium. For this purpose, BALB/c mice were divided into five groups (eight animals per group): (1) control group receiving sterile milk (non-fermented milk) and then challenged with *S.* Typhimurium—CTL; (2) experimental group receiving milk fermented by *L. rhamnosus* D1 for 7 days and then challenged with *S.* Typhimurium—D1(7d); (3) experimental group receiving milk fermented by *L. rhamnosus* D1 for 15 days and then challenged with *S.* Typhimurium—D1(15d); (4) experimental group receiving milk fermented by *L. rhamnosus* D1 inactivated by pasteurization (75 °C for 15 s) for 7 days and then challenged with *S.* Typhimurium—D1(In); (5) experimental group receiving milk fermented by *L. rhamnosus* D1 inactivated by sterilization (121 °C for 15 min) for 7 days and then challenged with *S.* Typhimurium—D1(SN). For 14 days post-challenge, survival was evaluated. This assay was repeated once.

In a second set of experiments (Disease Assay), we evaluated the protective effect of milk fermented by D1 throughout the course of the disease (5 days after infection). For this, BALB/c mice were divided into four groups (eight animals per group): (1) control group without treatment, drinking only sterile milk (NT); (2) control group receiving only milk fermented by *L. rhamnosus* D1 (D1); (3) control group receiving sterile milk for 7 days and then challenged with *S.* Typhimurium, being euthanized in 5th day after challenge (CTL); and (4) experimental group receiving milk fermented by *L. rhamnosus* D1 for 7 days and then challenged with *S.* Typhimurium, being euthanized in 5th day after challenge (D1(7d)). In challenged groups (D1(7d) and CTL), mice drank fermented milk for 7 days before the challenge and 5 days after the challenge. In D1 and NT groups, mice drank, respectively, fermented or non-fermented milk (sterile milk) for 12 days. During the 5 days after challenge, body weight evolution was assessed. After the experimental period, liver, ileum and ileal content were aseptically collected from euthanized mice and were used in hepatic bacterial translocation and histopathological assays, cytokine and defensin mRNA expression analyses and microbiota composition studies, respectively. This assay was repeated once.

### 2.4. Determination of Salmonella Translocation and Hepatic Inflammatory Foci

Liver samples from each mouse in the Disease Assay were weighted and then homogenized in sterile phosphate-buffered saline (PBS, 1:10, *w*/*v*). Serial decimal dilutions were prepared and then plated onto MacConkey agar (Merck-Millipore, Darmstadt, Germany) for a *Salmonella* specific count after incubation for 24 h at 37 °C. Only colorless colonies (lactose-negative) were considered. Translocation was expressed as log_10_ CFU/g of organ.

For hepatic histopathological analyses, liver samples were processed routinely for paraffin embedding and submitted to a microtome to obtain histological slides 4 mm thick. The slides were stained with hematoxylin and eosin (HE), coded and examined under an Olympus BX40 microscope (Olympus, Tokyo, Japan) by a single pathologist who was unaware of the experimental conditions of each group, and images were obtained with a Spot Insight Color (SPOT Imaging Solutions, Sterling Heights, MI, USA) with the image acquisition SPOT software (version 3.4.5; SPOT Imaging Solutions). For morphometric examination, obtained images were analyzed using ImageJ 1.48v software from NIH (Bethesda, MD, USA). Inflammatory foci were analyzed to assess hepatic tissue damage index, an accumulation of more than ten inflammatory cells being considered as an inflammatory focus [11]. Scoring was performed according to the following parameters: 0 (no inflammatory foci); 1 (discrete presence of inflammatory foci, <2 per field); 2 (moderate presence of inflammatory foci, 2–4 per field); and 3 (vigorous presence of inflammatory foci, >4 per field).

### 2.5. Relative Expression of Cytokines and Antimicrobial Peptides in Ileum

Small fragments (1 cm approximately) of proximal and distal ileum were collected from mice in the Disease Assay and stored in RNAlater (Ambion, Austin, TX, USA) at −80 °C until RNA extraction, which was conducted according to the TRIzol^®^ Reagent’s (Ambion, Austin, TX, USA) RNA isolation procedure. Samples were then treated with TurboDNA-freeKit^®^ (Ambion, Austin, TX, USA), according to the manufacturer’s instructions for DNA removal. cDNA of each sample was produced with High-Capacity cDNA Reverse Transcription kit (Applied Biosystems, Foster City, CA, USA), according to its manual instructions. Quantitative PCR (qPCR) was performed using QuantiTect SYBR green PCR kit (Qiagen, Hilden, Germany) and gene-specific primers for IFN-γ, IL-5, IL-6, IL-10, IL-12, TGF-β, Reg3β, Reg3γ and Lcn2, as well as housekeeping genes β-actin and GAPDH [11,20,27,28,29] (Supplemental Material File S1, Primer List). Amplification reactions were performed in a final volume of 20 μL, using 10 μL of SYBR green master mix and 50 ng of cDNA. Expression levels in the control group (without treatment) were used as calibration data. Results are shown graphically as fold changes in gene expression, using the means and standard deviations of the target cytokine expression amount (2^−ΔΔCT^) [30].

### 2.6. Ileum Microbiota Screening Through 16S rDNA Gene Sequencing

Total DNA from ileum contents was extracted with DNeasy PowerSoil Pro Kit (Qiagen, Hilden, Germany). Samples were sequenced on Illumina’s MiSeq^®^ platform, where the 16S V4 (250 bp) region was evaluated. Initially, a PCR reaction for the 16S rRNA gene was conducted with Phusion High Fidelity DNA^®^ Polymerase (New England Biolabs, Ipswich, MA, USA) for each sample, using barcoded primers. Reaction efficiencies were confirmed in a 1% agarose gel run with GelRed^®^ (Biotium, Freemont, CA, USA). Samples were then purified and standardized with SequalPrep Normalization Plate kit^®^ (Invitrogen, Carlsbad, CA, USA). A pool of all samples was then submitted to a new run on 1% agarose gel, and a 250 bp band was cut and purified with NucleoSpin Gel and PCR Clean-Up Kit^®^ (MachereyNagel, Düren, Germany). The final library was standardized at 2 nmol with QuBit^®^ 3.0 Fluorometer (Invitrogen, Carlsbad, CA, USA). The Kapa Library Quantification for NGS Kit^®^ (Kapa Biosystems, Wilmington, DE, USA) was used to assess the final library concentration. Finally, the library was loaded on Illumina’s MiSeq^®^ after preparation with MiSeq Reagent V2 Kit^®^ (Illumina, San Diego, CA, USA). Data obtained were analyzed with DADA2 and R using the Phyloseq package (v 1.16.2), with scripts previously designed by ENdEMIC group from Antwerp, Belgium.

### 2.7. Statistical Analysis

Differences between results (weight change, hepatic translocation, inflammatory foci, cytokines and antimicrobial peptides expression) were evaluated by analysis of variance (one-way ANOVA) with Tukey’s post-test (for comparison of three or more experimental groups) or Student’s *t*-test (for comparison of two experimental groups). For weight evolution, two-way ANOVA with Sidak’s post-test was used. Log-rank (Mantel–Cox) test was applied for the survival test and comparison between groups. All tests were performed using GraphPad Prism v. 8.4.3 for Windows from GraphPad Software (San Diego, CA, USA). Only results with *p* < 0.05 or lower were considered significant.

## 3. Results

### 3.1. Consumption of Milk Fermented by L. rhmanosus D1 Reduced the Mortality Rate of Mice Infected with S. Typhimurium

**Figure 1:**
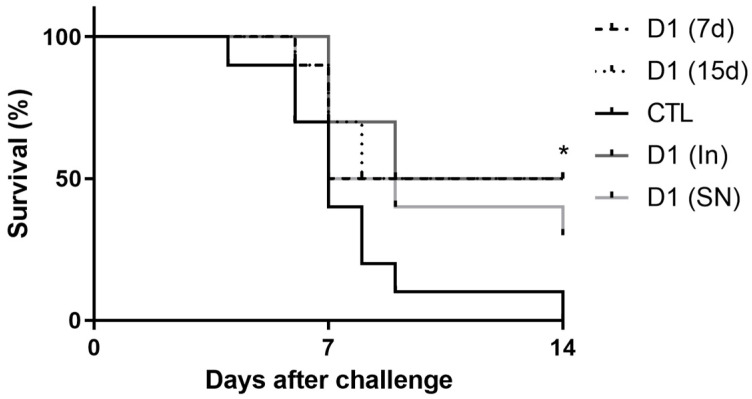
Mice treated with fermented milk (D1(7d) or D1(15d)) or pasteurized fermented milk (D1(In)) and challenged with *S.* Typhimurium had higher survival rate compared to mice treated with sterile milk, CTL. Challenge was conducted with 10^6^ CFU/mL of *S.* Typhimurium. D1(7d): BALB/c mice treated with milk fermented by *L. rhamnosus* D1 (10^8^ CFU/mL) for 7 days prior to challenge and during challenge (5 days); D1(15d): BALB/c mice treated with milk fermented by *L. rhamnosus* D1 (10^8^ CFU/mL) 15 days prior to challenge and during challenge; D1(In): BALB/c mice treated with inactivated fermented milk (by fast pasteurization −75 °C for 15 s) for 7 days prior to challenge and during challenge; D1(SN): BALB/c mice treated with inactivated fermented milk (by sterilization; 121 °C for 15 min) for 7 days prior to challenge and during challenge; CTL: BALB/c mice treated with sterile milk for 7 days prior to challenge and during challenge. * *p* < 0.05 by Log-rank (Mantel–Cox). *n* = 8 per group.

In the Mortality Assay, we evaluated the protective effect of milk fermented by *L. rhamnosus* D1 on the mortality rate of mice infected with *S.* Typhimurium. For this purpose, we used fermented milk containing viable cells (D1(7d) or D1(15d)), or containing cells killed by pasteurization (D1(In)) or sterilization (D1(SN)). As show in Figure 1, the administration of fermented milk for 15 or 7 days (D1(15d) or (D1(7d)) was able to significantly reduce the mortality of challenged mice compared to those that only drank sterile milk (non-fermented milk) and were challenged by *S.* Typhimurium (CTL, *p* > 0.05). Furthermore, we observed that pasteurized fermented milk (D1(In)) also had the same protective effect compared to the control group (CTL, *p* > 0.05).

### 3.2. Consumption of Milk Fermented by L. rhmanosus D1 Reduced Disease Severity in Infected Mice Through Epithelial Barrier Maintenance and Modulation of Cytokines and Antimicrobial Peptides in Ileum

**Figure 2:**
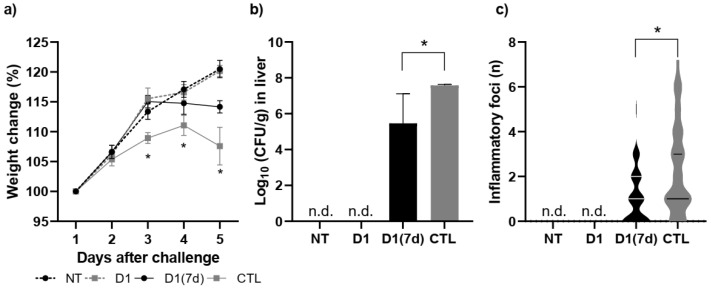
Mice treated with fermented milk (D1(7d)) and challenged with *S.* Typhimurium showed less (**a**) weight loss, (**b**) hepatic translocation, (**c**) and liver damage when compared to mice that were only challenged (CTL, treated with non-fermented milk). D1(7d): BALB/c mice treated with milk fermented by *L. rhamnosus* D1 (10^8^ CFU/mL) for 7 days prior to challenge and during challenge (5 days); CTL: BALB/c mice treated with sterile milk for 7 days prior to challenge and during challenge. Challenge was conducted with 10^6^ CFU/mL of *S.* Typhimurium. In weight change assay, * *p* < 0.01 by two-way ANOVA with Sidak’s post-test (D1(7d)xCTL). In translocation assay, * *p* < 0.01 using parametric Student’s *t*-test. In inflammatory foci assay, *p* < 0.05 using Mann–Whitney Student’s *t*-test (non-parametric). *n* = 8 per group. n.d. not detected.

In the Disease Assay, we aimed to demonstrate the mechanisms by which the consumption of fermented milk would be associated with the protective effect against infection by *S.* Typhimurium. For this, only D1(7d) was selected, as its performance was similar to that of D1(15d) in the Mortality Assay. Furthermore, since the CTL group had multiple deaths after 5 days of challenge, the comparison was performed on the 5th day after infection by *S.* Typhimurium. Therefore, we conducted this assay by administering the fermented milk containing viable cells for 7 days prior to the challenge with *S.* Typhimurium, with mice being euthanized 5 days after infection. As shown in Figure 2, mice treated with fermented milk (D1(7d)) and challenged with *S.* Typhimurium showed more (a) weight gain and less (b) hepatic translocation and (c) liver damage compared to mice that were only challenged (CTL, treated with non-fermented milk), indicating a protective effect (*p* < 0.05).

**Figure 3:**
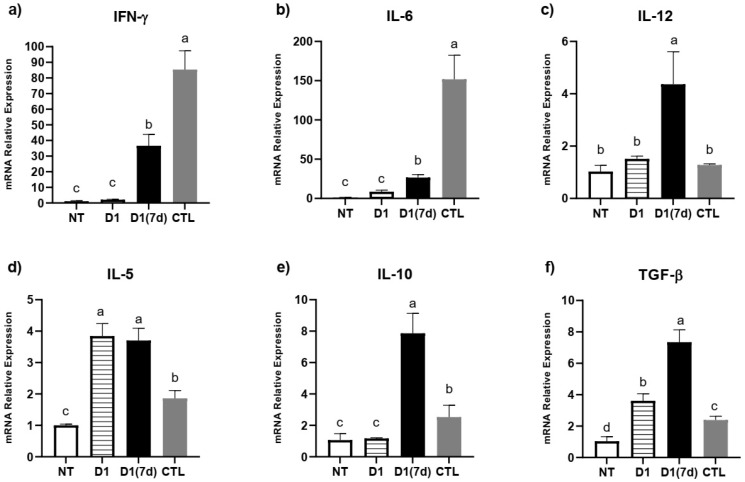
Immunomodulation triggered by ingestion of milk fermented by *L. rhamnosus* D1: (**a**) IFN-γ; (**b**) IL-6; (**c**) IL-12; (**d**) IL-5; (**e**) IL-10; (**f**) TGF-β. NT: BALB/c mice treated with sterile milk for 12 days; D1: BALB/c mice treated with milk fermented by *L. rhamnosus* D1 (10^8^ CFU/mL) for 12 days; D1(7d): BALB/c mice treated with milk fermented by *L. rhamnosus* D1 (10^8^ CFU/mL) during 7 days prior to challenge and during challenge (5 days); CTL: BALB/c mice treated with sterile milk for 7 days prior to challenge and during challenge. Challenge was conducted with 10^6^ CFU/mL of *S.* Typhimurium. Different letters represent *p* < 0.05 by one-way ANOVA with Tukey’s post-test. *n* = 3 per group.

The immunomodulation triggered by the ingestion of fermented milk was locally evaluated in the ileum, where the relative expression levels of IFN-γ, IL-5, IL-6, IL-10, IL-12 and TGF-β were evaluated (Figure 3). In this assay, challenged mice treated with milk fermented by *L. rhamnosus* D1 (D1(7d)) had a reduced expression of IFN-γ, IL-6 and IL-12, in addition to an increased expression of IL-5, IL-10 and TGF-β compared to those challenged with *S.* Typhimurium alone (CTL, *p* < 0.05).

**Figure 4:**
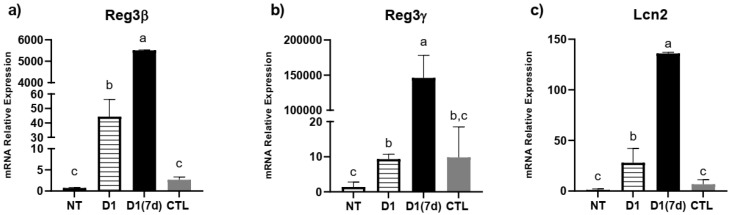
Fermented milk ingestion modulated antimicrobial peptide expression in ileum of both infected and non-infected mice: (**a**) Reg3β; (**b**) Reg3γ; (**c**) Lcn2. NT: BALB/c mice treated with sterile milk for 12 days; D1: BALB/c mice treated with milk fermented by *L. rhamnosus* D1 (10^8^ CFU/mL) for 12 days; D1(7d): BALB/c mice treated with milk fermented by *L. rhamnosus* D1 (10^8^ CFU/mL) during 7 days prior to challenge and during challenge (5 days); CTL: BALB/c mice treated with sterile milk for 7 days prior to challenge and during challenge. Challenge was conducted with 10^6^ CFU/mL of *S.* Typhimurium. Different letters represent *p* < 0.05 by one-way ANOVA with Tukey’s post-test. *n* = 3 per group.

Regarding antimicrobial peptides, we observed that the ingestion of fermented milk was able to stimulate the expression of Reg3β and Lcn2, regardless of the challenge with *S.* Typhimurium (D1 group, *p* < 0.05, Figure 4). In mice that were both treated and challenged (D1(7d) group), the expression of Reg3β, Reg3γ and Lncn2 was even more pronounced compared to mice that were only challenged (CTL, *p* < 0.05), suggesting that protection against infection may be related to the expression of these molecules (Figure 4).

### 3.3. Consumption of Milk Fermented by L. rhmanosus D1 Changed Ileum Microbiota in Infected Mice

**Figure 5:**
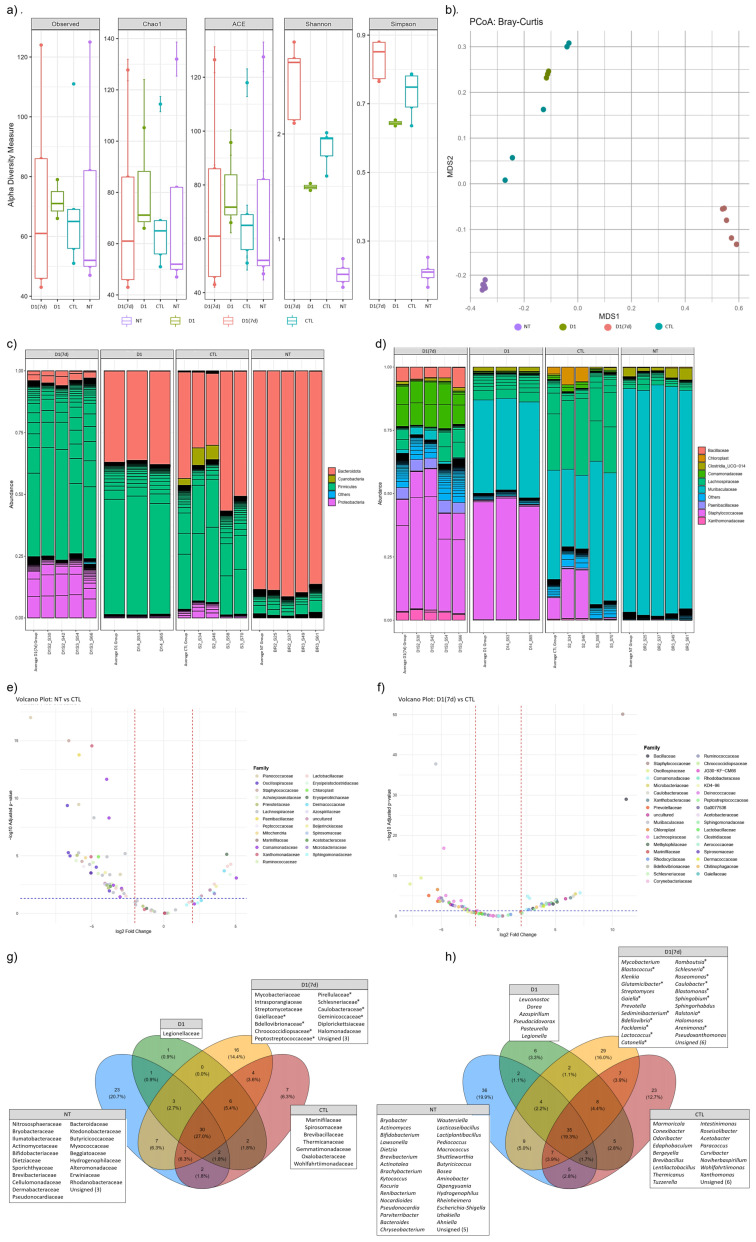
Fermented milk ingestion changed ileum microbiota of both infected and non-infected mice: (**a**) α-Diversity was measured using Chao1, ACE, Shannon and Simpson indices, and (**b**) β-Diversity index was calculated using Principal Coordinates Analysis (PCoA) based on Bray–Curtis dissimilarities. Barplot showing the relative abundance [%] of the top 90 most abundant bacterial taxa at (**c**) Phylum and (**d**) Family level. Volcano Plot showing significant abundance differences (*p* < 0.05) at Family level between (**e**) NT and CTL mice, as well as (**f**) D1(7d) and CTL mice. Venn diagram depicting exclusive taxa at (**g**) Family and (**h**) Genus level for experimental groups. NT: BALB/c mice treated with sterile milk for 12 days; D1: BALB/c mice treated with milk fermented by *L. rhamnosus* D1 (10^8^ CFU/mL) for 12 days; D1 (7d): BALB/c mice treated with milk fermented by *L. rhamnosus* D1 (10^8^ CFU/mL) during 7 days prior to challenge and during challenge (5 days); CTL: BALB/c mice treated with sterile milk for 7 days prior to challenge and during challenge. Challenge was conducted with 10^6^ CFU/mL of *S.* Typhimurium. * in (**g**,**h**) refers to unique taxa in D1(7d) group that had a significant increase in abundance when compared with CTL group (D1(7D) vs. CTL).

In addition to interacting with the mucosal host, specific probiotic strains are able to modulate the gut microbiota by altering its composition. In the present study, species richness, as measured by the Chao1 and ACE indices, did not differ significantly between experimental groups. Despite the similarity in species richness, a significant difference was observed in species evenness and abundance among these groups, as indicated by the Shannon index (Figure 5a). Notably, mice in the D1(7d) group (infected and treated with fermented milk) exhibited a higher microbial diversity compared to the infected control group (CTL, *p* < 0.05). Furthermore, the beta diversity analysis revealed that the bacterial community composition in the ileal microbiota was influenced by the treatment type, with the D1(7d) group displaying a distinct microbial profile relative to the CTL group (Figure 5b).

The taxonomic composition analysis at the Phylum level revealed that infection with *S.* Typhimurium increased the relative abundance of Firmicutes (CTL vs. NT comparison). In contrast, treatment with the fermented milk resulted in an increased abundance of Proteobacteria (D1(7d) vs. CTL, Figure 5c). At the Family level, infection was associated with an increased abundance of Staphylococcaceae, Lachnospiraceae and Comamonadaceae (CTL vs. NT). The treatment with fermented milk further modulated the microbiota, leading to increased abundances of Xanthomonadaceae, Staphylococcaceae, Paenibacillaceae, Comamonadaceae and Bacillaceae, along with a reduction in Lachnospiraceae abundance (D1(7d) vs. CTL, Figure 5d).

The comparison of ileal microbiota between untreated (NT) and *S.* Typhimurium-infected mice (CTL) revealed significant alterations in the abundance of 89 bacterial genera, distributed across 41 Families (Figure 5e, Supplemental Table S1, *p* < 0.05). Among the most affected genera—those showing the most significant increases in relative abundance following infection—were *Sporosarcina*, *Staphylococcus*, *Vulcaniibacterium*, *Paenibacillus* and *Schlegelella*. In contrast, *Pediococcus*, *Lactiplantibacillus*, *Kytococcus*, *Erysipelatoclostridium* and *Aquabacterium* exhibited the most significant reductions in abundance (Supplemental Table S1, *p* < 0.05). In a subsequent comparison between infected mice treated with fermented milk (D1(7d)) and infected mice (CTL), 101 genera, distributed across 48 Families, were significantly altered in abundance (Figure 5f, Supplemental Table S2, *p* < 0.05). In this context, *Staphylococcus*, *Bacillus*, *Aquabacterium*, *Caulobacter* and *Schlegelella* were among the identified genera showing the most notable reduction in response to the intervention. Conversely, *Muribaculum*, *Roseburia*, *Intestinimonas*, *Tyzzerella* and *Acetatifactor* exhibited a significant increase in abundance following the treatment (Supplemental Table S2, *p* < 0.05).

In addition to evaluating shifts in microbial abundance, we also assessed whether the different treatments influenced the presence of unique taxa among the experimental groups. To this end, a Venn diagram analysis was performed, which revealed the presence of 23, 1, 16 and 7 unique taxa at the Family level in the NT, D1, D1(7d) and CTL groups, respectively (Figure 5g). Notably, Caulobacteraceae, Bdellovibrionaceae, Schlesneriaceae, Chroococcidiopsaceae, Peptostreptococcaceae, Gaiellaceae, Pirellulaceae and Geminicoccaceae were exclusively identified in the ileal microbiota of infected mice treated with fermented milk (D1(7d)), with significantly higher abundances when compared to the infected untreated group (CTL, Figure 5g). At the Genus level, 36, 6, 29 and 23 unique genera were identified, respectively, in NT, D1, D1(7d) and CTL groups (Figure 5h). Among the genera exclusively identified in the D1(7d) group, *Caulobacter*, *Bdellovibrio*, *Schlesneria*, *Romboutsia*, *Catonella*, *Sphingobium*, *Facklamia*, *Sediminibacterium*, *Gaiella*, *Blastomonas*, *Roseomonas*, *Lactococcus*, *Glutamicibacter*, *Blastococcus*, *Ralstonia* and *Arenimonas* stood out for their significantly higher abundances compared to those observed in the untreated infected mice (CTL, Figure 5h).

## 4. Discussion

Through humoral and non-humoral mechanisms, probiotics exert their beneficial roles by promoting health [13,19]. In this study, we evaluated the protective effect of the ingestion of milk fermented by *L. rhamnosus* D1 against infection caused by *S.* Typhimurium in mice, which resembles that generated by *S.* Typhi in humans [11]. Unlike *S.* Typhimurium, *S.* Typhi, a human obligatory pathogen that causes Typhoid fever, is normally unable to infect mice. The main difference lies in TLR11 expression, which occurs in mice but not in humans, and, for this reason, the use of *S.* Typhimurium is a model that mimics, with limitations, the burden of *S.* Typhi in humans, since it is a consolidated in vivo model for salmonellosis [31].

In this animal model, the infection process begins in ileum (Local Phase), where the bacterium establishes itself before breaking through the epithelial mucosal barrier, accessing the systemic circulation and, subsequently, colonizing various organs such as the liver and spleen (Systemic Phase) [11,12], this being the final stage before sepsis and death. Sepsis caused by *S.* Typhimurium is still not well understood. However, in murine models of sepsis induced by LPS, it is characterized by an excessive synthesis of inflammatory cytokines, such as IL-6, IL-12 and IFN-γ, endothelial damage and coagulation disorders [6,32]. This clinical condition resembles what we observed in our infection model, where the modulation of IL-6, IL-12 and IFN-γ is crucial for the survival of infected mice [11,22].

In this study, we observed that the consumption of fermented milk containing viable cells of *L. rhamnosus* D1 for 15 (D1(15d)) or 7 (D1(7d)) days prior to *S.* Typhimurium challenge was able to protect mice, reducing mortality by 50%. The same effect was observed in mice challenged and treated with pasteurized fermented milk (D1(In)), indicating that the growth of *L. rhamnosus* D1 in the dairy matrix may produce substances with protective effects against Typhoid fever infection. Zagato et al. (2014) had demonstrated, with *L. paracasei* CBA L74, that a probiotic supernatant was able to induce an anti-inflammatory status in Dendritic Cells when co-incubated with *S.* Typhimurium, which endorses our findings [33]. Dou et al. (2021) also demonstrated that metabolites of *L. casei* ATCC 393 have an intestinal protective effect through caspase/IL-1β pathway modulation [34], which can also help to explain the possible mechanisms underlying the observed results of milk fermented by D1 with inactivated cells. In all cases, these results indicated that, in some way, the treatment was able to alter the course of the disease, reducing the occurrence of sepsis and subsequent death of infected mice.

To understand the mechanisms potentially associated with protection and mortality reduction, we analyzed mice treated for 7 days with fermented milk containing viable *L. rhamnosus* D1 cells and challenged with *S.* Typhimurium, over a period of 5 days post-infection (D1(7d)). In this regard, we observed that the treatment promoted animal health by reducing weight loss, as well as bacterial translocation and the number of inflammatory foci in the liver (hepatic injury), indirectly suggesting that the ileal epithelial barrier remained less damaged. These outcomes are consistent with earlier findings that certain probiotic strains can reinforce the intestinal barrier, thus reducing bacterial translocation to systemic organs [35]. Specifically, *L. rhamnosus* has been shown to enhance tight junction integrity and promote mucin production, both of which are crucial for limiting pathogen invasion [36,37,38]. However, whether the consumption of milk fermented by *L. rhamnosus* D1 altered the expression of these molecules still needs to be further investigated.

When we performed immunomodulation assays through qPCR, we found that the infected mice treated with fermented milk (D1(7d)) exhibited a reduced IL-6 and IFN-γ expression, which, as mentioned above, is associated with the process of sepsis and mortality in these animals [6,32]. Moreover, we observed an increased IL-10 and TGF-β expression, which are associated with a regulatory T cell response, whose role is to modulate T cell proliferation and the synthesis of inflammatory cytokines. In this context, the upregulation of these cytokines must have contributed to regulating inflammatory responses in ileum mucosa, reducing the expression of IL-6 and IFN-γ [14,21]. The dampening of IFN-γ, a key mediator in the immune response against intracellular pathogens like *S.* Typhimurium, must be interpreted cautiously; while necessary for pathogen clearance, excessive IFN-γ-driven inflammation can exacerbate tissue injury [19,39]. Therefore, the observed downregulation appears to reflect a well-regulated immune modulation, rather than suppression, and was, probably, trigged by an upregulation of IL-10 and TGF-β. In summary, one mechanism by which the consumption of milk fermented by *L. rhamnosus* D1 would protect mice in this model may be associated with an immunobiotic effect, through the modulation of pro- and anti-inflammatory cytokines. The literature reports several recent examples about the immunobiotic effects of probiotic strains in the context of salmonellosis, such as Liu et al. (2023) and Junaid et al. (2024), who, respectively, treated infected mice with *Lactiplantibacillus plantarum* Lp01 and *Lactobacillus acidophilus* (1.3251), and observed that the protective effect was associated with a reduced IL-6 and an increased IL-10 expression [14,21].

Another mechanism by which probiotics may exert their beneficial effects involves stimulating the production of antimicrobial peptides by the host’s mucosa [35]. These molecules are components of the innate immune defense in the gut, functioning both to inhibit pathogen colonization and to shape microbial community structure [23,24,25,40]. Reg3β and Reg3γ are peptides primarily produced by Paneth cells and enterocytes in the small intestine, and act on bacterial cell walls, leading to bacterial death [23,25]. Recent studies have shown that these peptides help to strengthen the intestinal epithelial barrier by promoting a physical separation between the microbiota and the host, thus preventing exacerbated mucosal immune responses [35,38]. The consumption of milk fermented by *L. rhamnosus* D1 by both challenged (D1(7d)) and unchallenged (D1) mice stimulated the expression of these two molecules, suggesting that they may be related to mouse protection, triggering a baseline priming effect on the host defense mechanisms, preventing an excessive microbiota leakage during infection and, consequently, reducing translocation and liver colonization. In this context, a lower translocation indicates that mice did not experience an exacerbated systemic inflammatory response, thereby avoiding the development of sepsis, which could ultimately lead to death [11,38]. Thus, one of the beneficial effects of fermented milk consumption was related to the reinforcement of the intestinal epithelial barrier through the increased expression of Reg3β and Reg3γ. In addition to these two molecules, we also evaluated the expression of Lcn2, whose bacteriostatic mechanism involves iron sequestering, inhibiting bacterial growth through this nutrient limitation [40,41]. In this direction, we observed that treated and challenged mice (D1(7d)) showed an increased expression of Lcn2, which also contributed to strengthening the epithelial barrier. Taken together, these data suggest that the protective effect of milk fermented by *L. rhamnosus* D1 involves a combination of immune modulation and a direct enhancement of the host barrier defenses.

In addition to interacting with the host, certain probiotic strains have the capacity to modulate the gut microbiota by altering its composition. In this study, we observed that the different treatments significantly affected the relative abundance of several microbial taxa. Many of these taxa have not yet been thoroughly investigated, and their roles in maintaining a healthy microbiota or contributing to dysbiosis—particularly in the context of *S.* Typhimurium infection—remain unclear. Consequently, data regarding these taxa will not be discussed in detail. Conversely, several other taxa identified in this study have previously been associated with intestinal health across various animal models of dysbiosis, whether of infectious or non-infectious origin. Here, we found that infection (CTL) led to an increased abundance of Phylum Firmicutes, as well as the Family Lachnospiraceae—both of which have been linked to chronic infections caused by Enterobacteriaceae, including members of *Salmonella* and *Escherichia* [42,43]. At the Genus level, infection was associated with an increase in *Paenibacillus* and a decrease in *Pediococcus*, *Lactiplantibacillus* and *Erysipelatoclostridium*. Recent studies have explored the probiotic potential of *Paenibacillus* strains, including their capacity to protect their hosts against enterobacterial infections [44,45]. Therefore, an increased abundance of this Genus could reflect a host-mediated attempt to restore microbial balance and mitigate dysbiosis. In contrast, *Pediococcus* and *Lactiplantibacillus* are widely recognized as safe, with multiple strains showing a supportive gut homeostasis and conferring protection against *S.* enterica infections [14,16,46,47,48]. Thus, the reduction in these taxa may be indicative of disease progression and microbial imbalance in the infected mice.

When treated with milk fermented by *L. rhamnosus* D1, the infected mice exhibited an increased relative abundance of Phylum Proteobacteria, whose overrepresentation has been widely recognized as a hallmark of dysbiosis associated with gut inflammation [49]. However, recent studies have shown that the prolonged administration of probiotic strains can modulate gut microbiota in this direction without necessarily impairing intestinal health [15]. At the Family level, we observed an increased abundance of Paenibacillaceae and Bacillaceae; some strains belonging to these taxa have been linked gut health, including in the context of intestinal inflammation triggered by enterobacterial infection [44,45,50,51]. At the Genus level, treatment of the infection significantly increased the abundance of *Muribaculum*, *Roseburia* and *Intestinimonas*, all of which include strains with potential health-promoting features [52,53,54], including in the context of Typhoid fever and the prevention of intestinal colonization by *S.* Typhimurium [55]. Notably, *Bdellovibrio* and *Facklamia* were uniquely detected in infected animals treated with fermented milk and drew particular attention. Members of *Bdellovibrio* are known predators of Gram-negative bacteria, including intestinal pathogens such as *E. coli* and *Salmonella* [56,57], while *Facklamia* has been associated with probiotic consumption in poultry [58], and may represent microbial signatures specifically favored by the fermented milk matrix or may result from competitive exclusion dynamics triggered by probiotic action. Altogether, these findings indicate that the consumption of the fermented milk changed the gut microbiota of infected mice, not only by modulating the existing taxa but also by facilitating the emergence of unique, potentially beneficial microbial lineages. Further studies are warranted to characterize the functional properties of these taxa and their contributions to host immunity, pathogen resistance and metabolic regulation. Moreover, metagenomic or metabolomic analyses could provide deeper insights into the functional consequences of these microbial shifts.

In summary, this study provides evidence that the consumption of milk fermented by *L. rhamnosus* D1 confers protection against *S.* Typhimurium infection in mice through multiple, potentially synergistic mechanisms—including the attenuation of systemic dissemination, preservation of tissue integrity, immunomodulation, upregulation of antimicrobial peptides and ileal microbiota modulation.

## 5. Conclusions

The present study demonstrates that milk fermented by *L. rhamnosus* D1 exerts a protective effect against *S.* Typhimurium infection in a murine model of Typhoid fever, significantly reducing mortality and mitigating clinical signs associated with systemic inflammation and sepsis. This protection appears to result from a multifactorial mechanism involving immunomodulation—characterized by the downregulation of pro-inflammatory cytokines (IL-6 and IFN-γ) and the upregulation of regulatory mediators (IL-10 and TGF-β)—as well as the enhancement of innate defense pathways, including an increased expression of antimicrobial peptides (Reg3β, Reg3γ and Lcn2). Additionally, the consumption of fermented milk led to notable shifts in the gut microbiota composition, favoring the emergence of potentially beneficial taxa and the reduction in dysbiosis-associated patterns. Importantly, the protective effects were observed both in the presence of viable bacteria and in pasteurized preparations, suggesting that bioactive compounds produced during fermentation may contribute significantly to the observed outcomes. These findings highlight the immunobiotic potential of *L. rhamnosus* D1 and reinforce the therapeutic promise of fermented dairy matrices as complementary strategies for managing enteric infections, such as *S.* Typhi in humans. However, to achieve this, we still need to conduct studies in human subjects to assess whether its consumption is, indeed, beneficial in the context of human Typhoid fever.

## Data Availability

The original contributions presented in this study are included in the article/Supplementary Material. Further inquiries can be directed to the corresponding author.

## Supplementary Materials

The following supporting information can be downloaded at https://www.mdpi.com/article/10.3390/microorganisms13102348/s1, File S1: Primers used in this study; Table S1: ASV changed by infection (CTL vs. NT); Table S2: ASV changed by treatment (CTL vs. D1(7d)).
