# Supplementary material for: Lacticaseibacillus rhamnosus D1 Fermented Milk Confers Protection Against Typhoid Fever Through Immunomodulation and Gut Microbiota Regulation in Mice
Source: Microorganisms. 2025 Oct 14;13(10):2348. doi: 10.3390/microorganisms13102348 (PMC12565942; doi:10.3390/microorganisms13102348)
Supplement: Supplementary file 1 [file microorganisms-13-02348-s001.zip › Supplemental Material File S1_ Primer List.pdf]

Supplemental Material File S1, Primers used in this study

Target	Sequence (5`-3`)	Reference
IL-10	GCTCTTACTGACTGGCATGAG	10.1073/pnas.1519906113
	CGCAGCTCTAGGAGCATGTG	
IFN-γ	TCAAGTGGCATAGATGTGGAAGAA	10.1006/meth.2001.1261
	TGGCTCTGCAGGATTTTCATG	
IL-5	AGCACAGTGGTGAAAGAGACCTT	10.1006/meth.2001.1261
	TCCAATGCATAGCTGGTGATTT	
IL-6	GAGGATACCACTCCCAACAGACC	10.1016/j.foodres.2020.109741
	AAGTGCATCATCGTTGTTCATACA	
TGF-b	TGACGTCACTGGAGTTGTACGG	10.1006/meth.2001.1261
	GGTTCATGTCATGGATGGTGC	
IL-12	GGAAGCACGGCAGCAGAATA	10.1006/meth.2001.1261
	AACTTGAGGGAGAAGTAGGAATGG	
Reg3β	CTGCCTTAGACCGTGCTTTC	10.1371/journal.pone.0020749
	ATAGGGCAACTTCACCTCAC	
Reg3γ	CCTTCCTCTTCCTCAGGCAAT	10.1016/j.cell.2009.09.033
	TAATTCTCTCTCCACTTCAGAAATCCT	
Lcn2	ACATTTGTTCCAAGCTCCAGGGC	10.1016/j.chom.2013.06.007
	CATGGCGAACTGGTTGTAGTCCG	
β-actin	AGAGGGAAATCGTGCGTGAC	10.1007/s12602-020-09634-x
	CAATAGTGATGACCTGGCCGT	
GAPDH	TCACCACCATGGAGAAGGC	10.1016/j.foodres.2020.109741
	GCTAAGCAGTTGGTGGTGCA	
